# *In Vivo* ZIMIR Imaging of Mouse Pancreatic Islet Cells Shows Oscillatory Insulin Secretion

**DOI:** 10.3389/fendo.2021.613964

**Published:** 2021-03-09

**Authors:** Shiuhwei Chen, ZhiJiang Huang, Harrison Kidd, Min Kim, Eul Hyun Suh, Shangkui Xie, Ebrahim H. Ghazvini Zadeh, Yan Xu, A. Dean Sherry, Philipp E. Scherer, Wen-hong Li

**Affiliations:** ^1^Departments of Cell Biology and of Biochemistry, University of Texas Southwestern Medical, Dallas, TX, United States; ^2^Touchstone Diabetes Center, University of Texas Southwestern Medical Center, Dallas, TX, United States; ^3^Department of Cell Biology, University of Texas Southwestern Medical Center, Dallas, TX, United States; ^4^Department of Biological Sciences, School of Life Sciences, Ulsan National Institute of Science and Technology, Ulsan, South Korea; ^5^Advanced Imaging Research Center, University of Texas Southwestern Medical Center, Dallas, TX, United States; ^6^Department of Chemistry and Biochemistry, University of Texas Dallas, Richardson, TX, United States; ^7^Department of Radiology, University of Texas Southwestern Medical Center, Dallas, TX, United States

**Keywords:** insulin oscillation, ZIMIR, ZIGIR, HaloTag, intravital microscopy (IVM), beta cell mass, imaging insulin secretion, beta cell imaging

## Abstract

Appropriate insulin secretion is essential for maintaining euglycemia, and impairment or loss of insulin release represents a causal event leading to diabetes. There have been extensive efforts of studying insulin secretion and its regulation using a variety of biological preparations, yet it remains challenging to monitor the dynamics of insulin secretion at the cellular level in the intact pancreas of living animals, where islet cells are supplied with physiological blood circulation and oxygenation, nerve innervation, and tissue support of surrounding exocrine cells. Herein we presented our pilot efforts of ZIMIR imaging in pancreatic islet cells in a living mouse. The imaging tracked insulin/Zn^2+^ release of individual islet β-cells in the intact pancreas with high spatiotemporal resolution, revealing a rhythmic secretion activity that appeared to be synchronized among islet β-cells. To facilitate probe delivery to islet cells, we also developed a chemogenetic approach by expressing the HaloTag protein on the cell surface. Finally, we demonstrated the application of a fluorescent granule zinc indicator, ZIGIR, as a selective and efficient islet cell marker in living animals through systemic delivery. We expect future optimization and integration of these approaches would enable longitudinal tracking of beta cell mass and function *in vivo* by optical imaging.

## Introduction

The islet of Langerhans plays essential roles in controlling metabolism and glucose homeostasis through the release of peptide hormones. The islet beta cell, in particular, is crucial for maintaining euglycemia *via* insulin secretion. In healthy subjects, insulin secretion is tightly regulated, and beta cells release insulin in response to nutrient fluctuations to clamp blood glucose within a narrow range. There has been growing interests in characterizing insulin release dynamics and studying its physiological regulation *in vivo*. By sampling the total insulin output from the pancreas, a number of studies have revealed the dynamic feature of insulin release in live animals, including rodents, dogs, and human (1, 2).

At the cellular and subcellular level, our understanding of insulin secretion *in vivo* is still limited. The lack of imaging assays capable of tracking insulin release of single cells or individual islets in live animals remains to be a roadblock towards functional analysis of islet beta cells *in vivo* (3). Previously, we developed a fluorescent, cell-surface targeted zinc indicator for monitoring induced exocytotic release (ZIMIR) (4). Exploiting Zn^2+^ elevation at the cell surface as a surrogate marker of insulin release, we applied laser scanning confocal microscopy to image ZIMIR and to map the spatiotemporal characteristics of insulin release in isolated islets. Herein we report our efforts of extending ZIMIR imaging to living mice. We developed a surgery procedure to label islet cells with ZIMIR through the celiac artery. Confocal ZIMIR imaging revealed oscillatory and synchronized insulin release among islet beta cells in a living mouse. Moreover, to facilitate probe delivery to islet cells, we exploited the HaloTag labeling technology and developed a chemogenetic approach for the targeted probe delivery to the plasma membrane of beta cells. Finally, we presented data to demonstrate the utility of a recently developed granule Zn^2+^ indicator, ZIGIR, as a selective and efficient marker of islet beta cells *in vivo via* systemic delivery.

## Material and Methods

### Mouse Maintenance and Surgery

All protocols for mouse use and euthanasia were reviewed and approved by the Institutional Animal Care and Use Committee of the University of Texas Southwestern Medical Center. All mice, including C57Bl/6J, MIP-GFP (Jackson Laboratory stock No. 006864), MIP-DsRed (Jackson No. 006866), MIP-rtTA (5), TRE-pDisplay-HaloTag-Myc were maintained in 12-h dark/light cycles, with *ad libitum* access to diet (Teklad 2016) and water. Mice 10–15 weeks old were used for the experiments. The TRE-pDisplay-HaloTag-Myc mouse was generated by the UTSW transgenic mouse core facility by cloning the pDisplay-HaloTag-Myc sequence (6) downstream of a TRE vector (5). Sprague Dawley rats were from Charles River.

### Intravital Imaging of Exteriorized Pancreas in Mice

To image islets in the exteriorized mouse pancreas, we customized an imaging platform containing a flexible stand to facilitate accessing pancreatic islets, and a home-made stabilizer to constrain mouse movement. During image acquisition, animals were laid on top of a heating pad to maintain body temperature. The entire imaging platform was enclosed within a temperature and humidity-controlled chamber. The exteriorized pancreas of an anesthetized mouse was carefully placed on the imaging platform, and islets close to the pancreas surface were identified and centered beneath the objective. Vaseline was applied to the sides of pancreas, which was sandwiched between two pedals of the stabilizer. We then applied a vacuum grease (Dow Corning) to adhere the two pedals and to seal the opening of the top pedal with a No. 1 glass coverslip. A small volume of saline was placed on top of the glass coverslip, through which the islets underneath were imaged by a dipping lens (20x objective). To image islet blood flow, we injected Texas-Red labeled dextran (70 KDa, 0.2 mg in 0.1 ml DPBS) to a MIP-GFP mouse through a catheter installed at the jugular vein 3 days earlier. To test bolus dye loading of pancreatic cells, we micro-injected Cy3-C12 (20 µM) to the mouse pancreas under a dissection scope.

To label pancreatic islets with amphipathic dyes (Cy3-C12 or ZIMIR) through the splenic artery, we temporarily blocked blood circulation to the celiac artery with a micro vessel clip, and infused 0.2 ml of dye solution (20 µM of Cy3-C12, or 100 µM of ZIMIR in DPBS) through the splenic artery using a 31 gauge needle. We removed the micro vessel clip 5 min later to restore the blood circulation and started confocal imaging within the next 10–15 min. Prior to imaging the ZIMIR signal, the mouse received an infusion of dextrose (50% in water) through the jugular vein catheter for 30 s at a rate of 25 µl/min.

Confocal imaging was performed on an upright LSM510-Meta system (Zeiss). ZIMIR and GFP were excited with 488 nm laser and detected at 500–560 nm. Dextran-Texas Red and Cy3-C12 were excited with 561 nm laser and detected at 570–625 nm.

### HaloTag Labeling of Islet Beta Cells

Expression of HaloTag-Myc in islet beta cells was confirmed by immunofluorescence. After feeding a transgenic mouse (*MIP-rtTA::TRE-pDisplay-HaloTag-Myc*) with Dox200 chow (Envigo) for 1 week, we harvested the pancreas and prepared formalin fixed paraffin embedded (FFPE) tissue section (10 µm). The tissue section was labeled with antibodies against insulin (Agilent, A0564) and Myc (Upstate, 06-549), and stained with the corresponding secondary antibodies (Jackson Immunoresearch, 706-545-148 and 711-165-152, respectively). To label beta cells *in vivo via* systemic dye delivery, we injected Fluo-HaloTag-2 (40 µM x 0.2 ml) through the tail vein of a transgenic mouse that had been on Dox200 for a week. We then sacrificed the mouse 30 min later and performed transcardial perfusion with ice-cold PBS buffer for 10 min at a flow rate of 1 ml/min. The pancreas was harvested immediately afterwards. A piece of pancreatic tissue containing both islets and exocrine cells was micro-dissected and imaged by confocal microscopy (Ex 488 nm, Em 500–550 nm).

To label HEK293 cells with ZIMIR-HaloTag, we infected the cells with a pDisplay-HaloTag plasmid and Lipofectamine for 48 h. Cells were then washed with a secretion assay buffer (SAB) containing 114 mM NaCl, 4.7 mM KCl, 1.2 mM KH_2_PO_4_, 2.5 mM CaCl_2_, 1.16 mM MgSO_4_, 3 mM glucose, and 20 mM Hepes (pH 7.4). Cells were then incubated with ZIMIR-HaloTag (2 µM) in the SAB buffer for 30 min at RT, washed and imaged by epifluorescence (Zeiss Axiovert 200). A high Zn^2+^ buffer (1 μM free Zn^2+^, buffered by 8.1 mM l-histidine, 2.6 mM ZnO in 10 mM Hepes, 5 mM KCl, 140 mM NaCl, pH 7.4) was added at the end to enhance ZIMIR-HaloTag signal.

To label mouse islets with ZIMIR-HaloTag, we fed a transgenic mouse (*MIP-rtTA::TRE-pDisplay-HaloTag-Myc*) with Dox200 chow (Envigo) for 2 weeks. During the last 3 days of the 2-week feeding period, we gave the mouse 0.5 ml doxycycline (2 mg/ml) three times a day *via* oral gavage to boost doxycycline dosing and to further enhance HaloTag expression. We then isolated mouse islets following the standard protocol of collagenase digestion (4). The isolated mouse islets were cultured in RPMI medium for 2 h to allow recovery, and subsequently incubated with ZIMIR HaloTag (2 µM) in the SAB buffer (with 3 mM glucose, 10 µM EDTA and 2 µM DPAS (4)) for 30 min at room temperature. Islets were then washed once and imaged by confocal microscopy at either basal (3 mM) or elevated glucose (20 mM) in SAB.

### ZIGIR Labeling of Islets *In Vitro* and *In Vivo*

To label islet cells with ZIGIR *in vivo*, we injected a PBS solution (0.2 ml for mouse, 2 ml for rat) containing ZIGIR (400 nmol/Kg) *via* the tail vein. The animal was sacrificed 30 min later for the islet isolation. The isolated islets were imaged by confocal microscopy exciting at 561 nm (Em 570–620 nm). To label islets with ZIGIR *in vitro*, hand-picked mouse islets (C57Bl6/J) were incubated in the SAB solution with ZIGIR (1 µM, diluted from 1 mM stock in DMF) for 15 min at 37°C. Islets were then washed once with SAB and imaged by confocal microscopy.

## Results

### Imaging Platform

**Figure 1:**
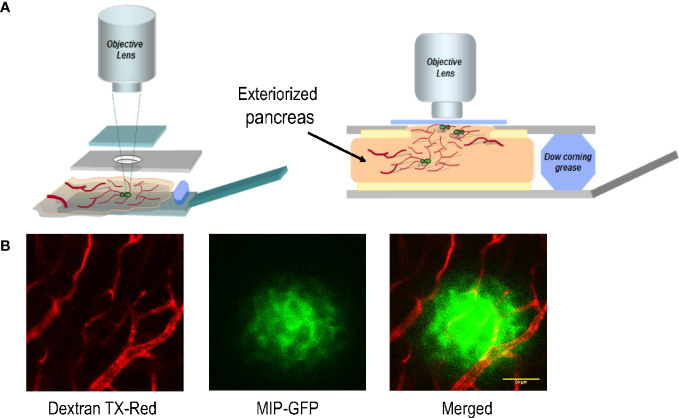
The platform for imaging islet cells in the intact pancreas of a live mouse. **(A)** Schematic of the customized imaging reservoir for housing the exteriorized pancreas for intravital imaging. **(B)** Example images showing blood vessels surrounding and within an islet of a MIP-GFP mouse. Dextran TX-Red was delivered through tail vein injection to label the vasculature.

To establish and optimize an imaging platform for monitoring islet cells in the intact pancreas, we implemented a minimally invasive surgical procedure for exteriorizing the mouse pancreas for intravital imaging. A similar procedure has been successfully applied to track immune cells during pancreas inflammation in a mouse model of type 1 diabetes (7). In this protocol, we surgically exposed the pancreas of a mouse under anesthesia, and placed it in a customized reservoir containing a heated physiological saline (**Figure 1**). The top of the reservoir was covered with a glass coverslip which was gently pressed against the tissue underneath.

### Dye Loading to Cells in the Pancreas *via* Microinjection

When applied to isolated islets, ZIMIR diffuses through interstitial space to label individual islet cells (4). To label islet cells with ZIMIR in the intact pancreas, we initially considered a multicell bolus loading method previously developed for loading fluorescent indicators to a population of cells in brain slices (8). The bolus loading involves injecting a concentrated DMSO dye stock solution into the tissue. The injected dye solution then diffuses to nearby areas, where the cells take up the dye.

**Figure 2:**
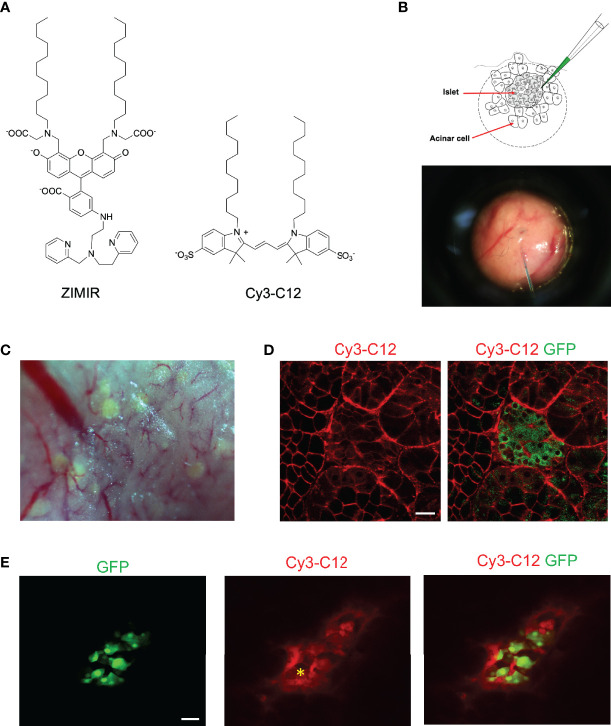
Labeling mouse islet cells in the intact pancreas *via* microinjection of dye solutions. **(A)** Chemical structures of ZIMIR and Cy3-C12. **(B)** Schematic of bolus loading of pancreatic cells *via* microinjection. The picture showed a needle filled with a dye solution piercing into the exocrine tissue near an islet. **(C)** A bright field image of the pancreatic tissue of a MIP-GFP mouse. Islets near the surface presented as light green puncta arising from GFP expression. **(D)** Confocal fluorescence images of a pancreas (MIP-GFP) post-injecting Cy3-C12 into the exocrine tissue. **(E)** Confocal fluorescence images of a pancreas (MIP-GFP) where an islet was injected with Cy3-C12. The yellow asterisk indicates the site of injection. Scale bar = 20 µm.

To adopt this method for labeling pancreatic islet cells, we applied an amphipathic fluorophore, Cy3-C12, to label islet cells in MIP-GFP mice. Cy3-C12, like ZIMIR, contains a pair of dodecyl alkyl chains for membrane anchoring (**Figure 2A**). Since it has two sulfonate anions and one quaternary ammonium cation, Cy3-C12 is impermeant to the cell membrane. It labels the outer leaflet of the plasma membrane similarly as ZIMIR. Cy3-C12 emits bright orange fluorescence, so it spectrally complements MIP-GFP expressed in the beta cell, enabling dual color imaging to assess dye labeling of the islet cell after dye injection.

After locating an islet near the surface of the exteriorized pancreas, we microinjected Cy3-C12 into the exocrine tissue near the islet (**Figures 2B, C**). We then imaged dye distribution and cellular dye uptake by confocal microscopy. The injected Cy3-C12 yielded an intense membrane labeling of pancreatic acinar cells near the site of injection (**Figure 2D**). However, Cy3-C12 labeling within the islet was much dimmer, and it appeared to be largely restricted to the intra-islet capillaries. We attributed this weak islet labeling to the retardation of dye diffusion across the peri-islet capsule. The peri-islet capsule was thought to function as a barrier between the endocrine and exocrine compartments (9, 10). To circumvent the problem, we attempted to deliver the dye to islet cells by positioning the injection needle within the islet. The intra-islet injection did confine the dye delivery to the islet (**Figure 2E**). However, unlike the membrane-specific labeling observed in the acinar cells (**Figure 2D**), we observed promiscuous distribution of Cy3-C12 throughout islet cells with a prominent intracellular staining.

### Dye Loading of Islet Cells *via* Pancreas Perfusion Through the Splenic Artery

**Figure 3:**
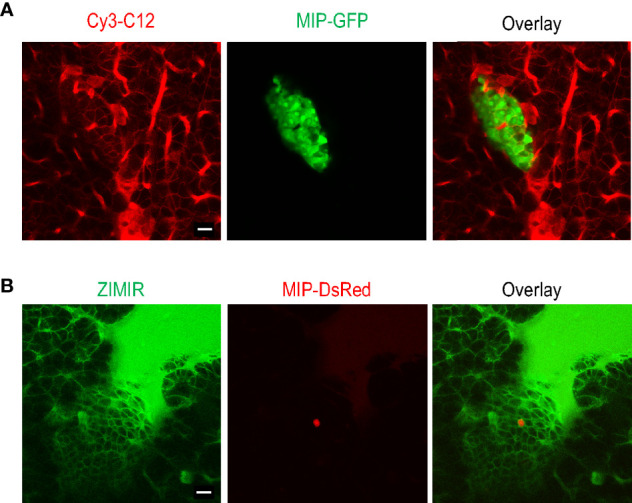
Labeling mouse islet cells in the intact pancreas *via* dye infusion through the splenic artery. **(A)** Confocal fluorescence images of a pancreas (MIP-GFP) post-infusing Cy3-C12 for 5 min into the exocrine tissue. **(B)** Confocal fluorescence images of a pancreas (MIP-DsRed) post-infusing ZIMIR. The scarce DsRed labeling was likely due to the low penetrance of MIP-DsRed transgene in this mouse. Scale bar = 20 µm.

Given the obstacles encountered with the bolus loading of pancreatic islet cells, we explored an alternative approach of delivering small amphipathic dyes to the islet. The splenic artery is a branch of celiac artery that supplies blood to the neck, body and tail regions of the pancreas. After exteriorizing a pancreas, we blocked blood flow of the celiac artery briefly by clamping the vessel with a micro-clip. We then infused a small volume of Cy3-C12 solution (0.2 ml) through the splenic artery to deliver the dye to the pancreas. After 5 min, we removed the micro-clip to restore the circulation. Confocal imaging of the dye-perfused pancreas confirmed that the plasma membranes of both endocrine and exocrine cells were labeled with Cy3-C12 (**Figure 3A**). Similarly, delivery of ZIMIR solution through the splenic artery also labeled pancreatic islet cells (**Figure 3B**). Hence, this dye infusion procedure through the vasculature yielded sufficient labeling of both endocrine and exocrine pancreatic cells for imaging applications.

### Intravital ZIMIR Imaging Revealed Oscillatory Insulin/Zn^2+^ Secretion *In Vivo*

**Figure 4:**
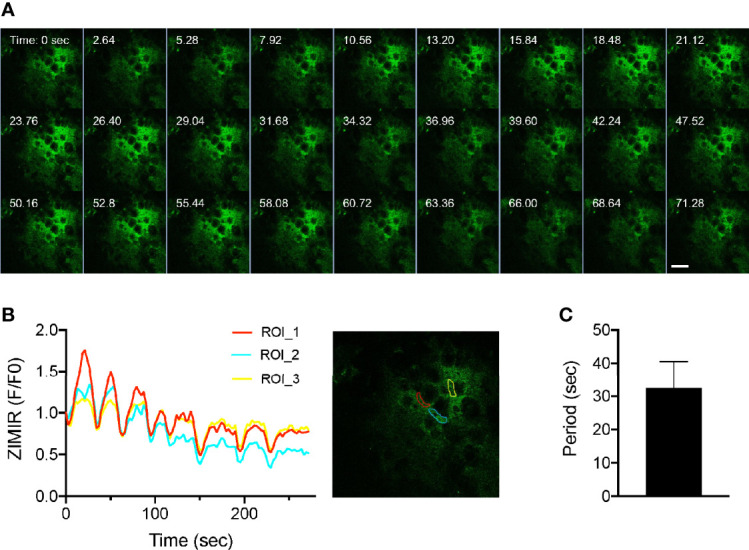
Intravital ZIMIR imaging showed oscillatory insulin/Zn^2+^ release of mouse islet cells. **(A)** Time lapse confocal ZIMIR images of pancreatic islet cells of a mouse under anesthesia. The mouse received a glucose injection just before the imaging session. Scale bar = 20 µm. **(B)** Time course of ZIMIR signal in three regions of interest (ROI) as indicated in the image to the right. **(C)** Average oscillation period of insulin/Zn^2+^ release (mean ± stdev) calculated from the time course shown in **(B)**.

Having established the imaging platform and a procedure for labeling islet cells in the intact pancreas, we applied the method to image insulin secretion in a living mouse. After labeling a mouse pancreas with ZIMIR through the splenic artery, we located an islet near the surface and centered it below the objective. We then challenged the mouse with a bolus of glucose through the jugular vein infusion. Subsequent confocal ZIMIR imaging showed a highly rhythmic insulin/Zn^2+^ secretion among a cluster of neighboring cells (**Figure 4**). Quantification of cell surface ZIMIR signal established a time course and an oscillation period of 32.48 ± 8.06 s (mean ± stdev).

### Targeted Dye Delivery to Islet Beta Cells *In Vivo* Through HaloTag Labeling

**Figure 5:**
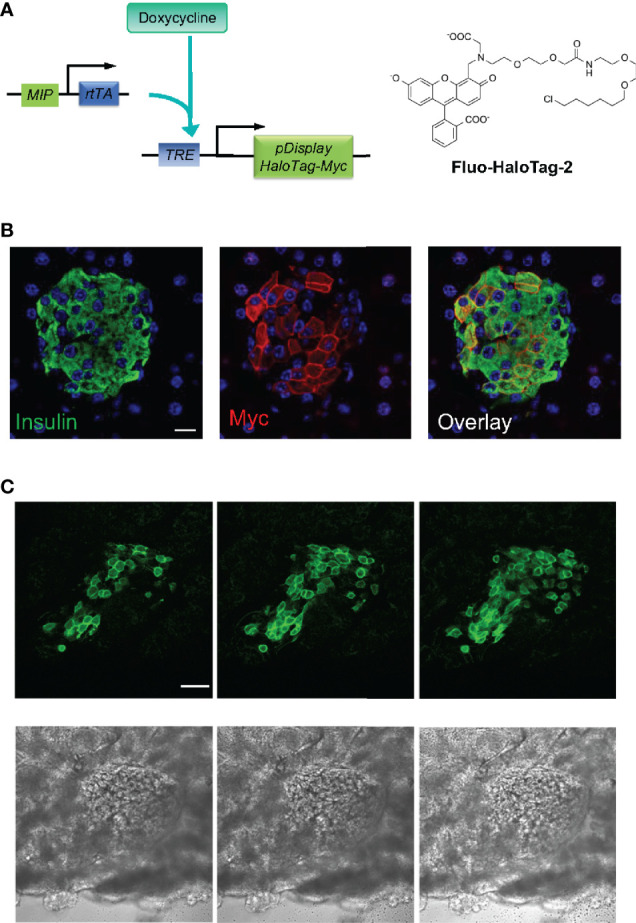
*In vivo* dye labeling of islet beta cells expressing HaloTag. **(A)** Transgenic mice harboring two transgenes express the HaloTag-Myc protein in beta cells upon doxycycline (Dox) induction. HaloTag labels itself by reacting with its ligands such as Fluo HaloTag-2. **(B)** Confirmation of HaloTag-Myc protein expression in islet beta cells by immunofluorescence. The pancreatic tissue section of a transgenic mouse (*MIP-rtTA::TRE-pDisplay-HaloTag-Myc*) fed on Dox200 for 1 week was stained with antibodies against insulin and Myc. Scale bar = 10 µm. **(C)** Confocal images of a dissected pancreas from a transgenic mouse fed with doxycycline for 1 week. The mouse received Fluo HaloTag-2 through tail vein injection 30 min before pancreas harvest. Three consecutive confocal sections 10 µm apart along the z-axis were shown. The bottom row shows the corresponding bright field images. Scale bar = 50 µm.

The success of capturing oscillatory insulin/Zn^2+^ secretion *in vivo* at the cellular resolution demonstrated the versatility and the potential of ZIMIR imaging. To facilitate labeling islet beta cells in live animals while minimizing animal stress, we explored an alternative strategy of targeted dye delivery through systemic circulation and tail vein injection. In this approach, we expressed a self-labeling enzyme, HaloTag (11), at the cell surface of islet beta cells. The HaloTag enzyme reacts with small molecules containing a haloalkane moiety to form a covalent bond between the protein itself and the small molecule. We bred a transgenic mouse that harbored two transgenes: *MIP-rtTA* and *TRE-pDisplay-HaloTag-Myc* (**Figure 5A**). *MIP-rtTA* drives the expression of reverse tetracycline transactivator (*rtTA*) in beta cells under the control of the mouse insulin promoter (*MIP*) (5). Addition of tetracycline activates the transactivator, which then binds to the tetracycline responsive element (*TRE*) to induce the expression of HaloTag protein selectively in beta cells. The HaloTag protein with a Myc tag was targeted to the cell surface by fusing with a PDGFR transmembrane domain (pDisplay construct) (6). After feeding a transgenic *MIP-rtTA::TRE-pDisplay-HaloTag-Myc* mouse doxycycline for a week, we confirmed the expression of HaloTag-Myc selectively at the beta cell surface by immunofluorescence (**Figure 5B**). Consistently, tail vein injection of a fluorescent HaloTag substrate, Fluo-HaloTag-2 (6), selectively labeled islet beta cells in a live transgenic mouse (**Figure 5C**). This *in vivo* labeling through systemic delivery of Fluo-HaloTag-2 was remarkably efficient, and it yielded a strong labeling of islet beta cells in just 30 min after tail vein injection of Fluo-HaloTag-2.

**Figure 6:**
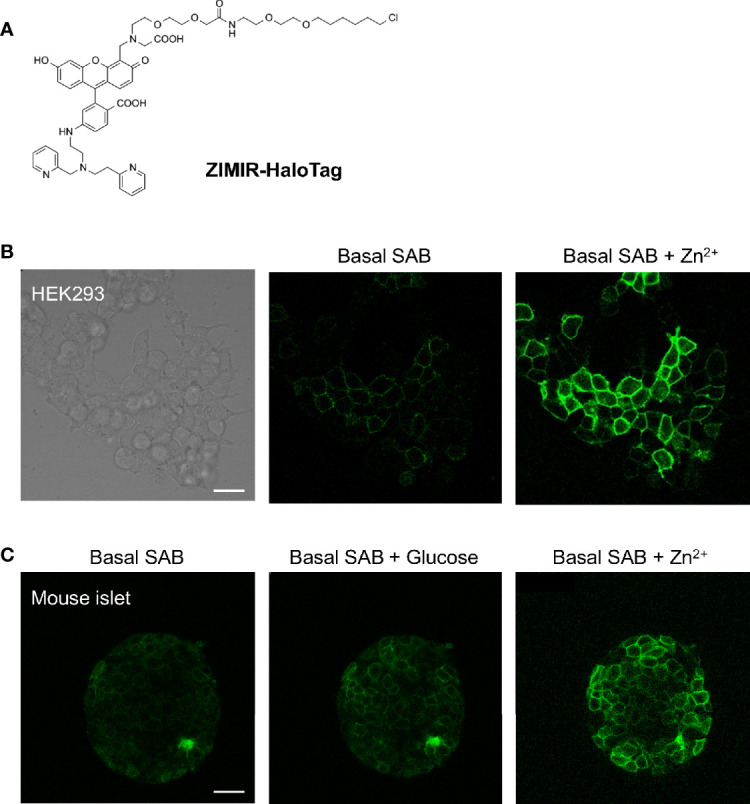
Imaging insulin/Zn^2+^ secretion in isolated mouse islets with ZIMIR-HaloTag. **(A)** Chemical structure of ZIMIR-HaloTag. **(B)** ZIMIR-HaloTag labeled the plasma membrane of HEK293 cells expressing pDisplay-HaloTag and responded to Zn^2+^ elevation in the medium. **(C)** Confocal imaging of insulin/Zn^2+^ secretion in an isolated islet from a MIP-rtTA::TRE-pDisplay-HaloTag-Myc mouse (doxycycline induced). The islet was labeled with ZIMIR-HaloTag (2 µM for 30 min) *in vitro*. After glucose challenge, a high Zn^2+^ buffer (1 µM Zn^2+^) was added. Scale bar = 20 µm.

Having established the feasibility of *in vivo* beta cell labeling using this chemogenetic approach, we went on to test ZIMIR-HaloTag for imaging insulin/Zn^2+^ release in beta cells expressing HaloTag. ZIMIR-HaloTag contains the same Zn^2+^ binding motif and fluorophore as ZIMIR (**Figure 6A**). It binds Zn^2+^ with an affinity of 0.13 µM and displays a 15-fold fluorescence enhancement upon Zn^2+^ binding (6). In cultured HEK293 cells infected with a pDisplay-HaloTag-Myc plasmid, ZIMIR-HaloTag labeled the cell plasma membrane and showed a robust fluorescence increase in response to Zn^2+^ elevation in the extracellular medium (**Figure 6B**). However, when we attempted to label islet beta cells with ZIMIR-HaloTag in a living transgenic mouse after doxycycline induction, we only observed a rather low fluorescence signal similar to the background intensity in beta cells following the same procedure as we had used for Fluo-HaloTag-2. After isolating mouse islets, we were nonetheless able to label the islet beta cells *in vitro* with ZIMIR-HaloTag (**Figure 6C**), confirming HaloTag expression in beta cells. Moreover, glucose stimulation promoted insulin/Zn^2+^ secretion and increased ZIMIR-HaloTag intensity in a number of regions in the islet, demonstrating an intact functionality of the isolated islets in glucose sensing and hormone release. Adding Zn^2+^ to the medium further raised ZIMIR-HaloTag intensity to a supramaximal level throughout the entire islet (**Figure 6C**).

### *In Vivo* Labeling of Dense Core Granules and Islet Cells With ZIGIR

**Figure 7:**
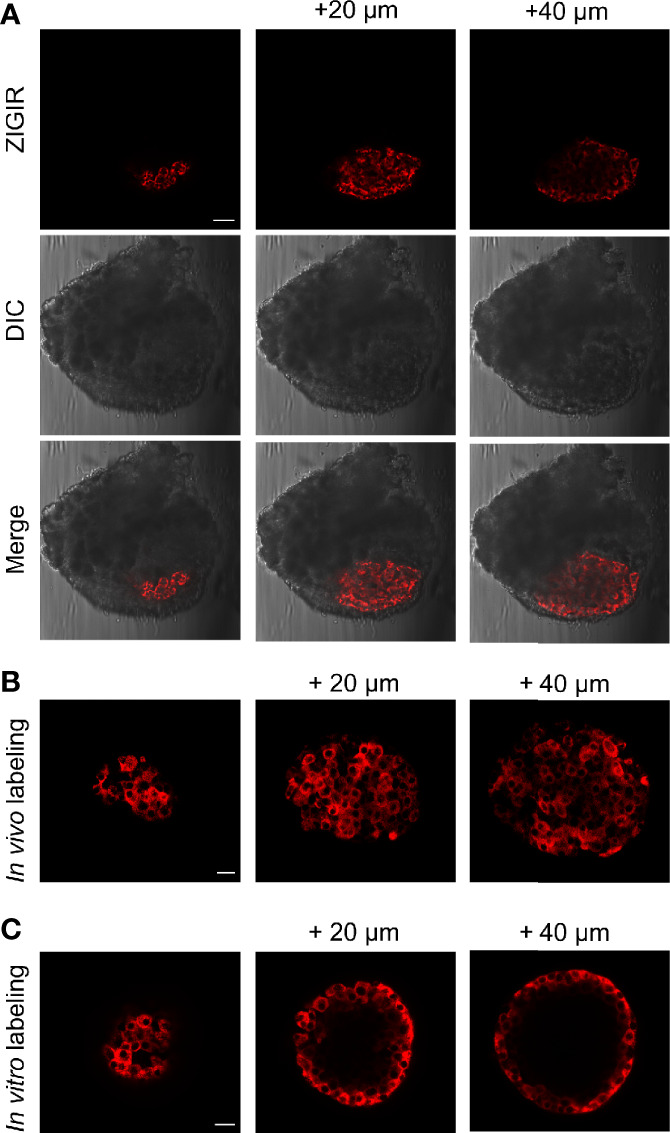
*In vivo* systemic delivery of ZIGIR selectively labeled pancreatic endocrine cells throughout entire islets. **(A)** ZIGIR selectively labeled rat islet cells after tail vein injection. Confocal images of a pancreatic tissue from a rat injected with ZIGIR. Three consecutive confocal sections 20 µm apart along the z-axis were shown. ZIGIR signal was seen only in the islet but not the exocrine cells. Scale bar = 100 µm. **(B, C)** ZIGIR labeled mouse islet cells throughout the islet after tail vein injection **(B)**, but only labeled the outer cell layer when it was applied to the isolated mouse islets *in vitro* **(C)**. Three consecutive confocal sections of ZIGIR-labeled islets were shown. Scale bar = 20 µm.

In parallel to our efforts of imaging beta cell function, we are also exploring new approaches for tagging native islet cells based on their intrinsic properties for evaluating islet cell mass. Recently, we have developed a zinc granule indicator, ZIGIR, as a specific, brightly fluorescent label of zinc rich secretory granules (12). ZIGIR is cell membrane permeable and accumulates in the dense core granules of islet cells, exhibiting the highest fluorescence intensity in the insulin granule owing to its high Zn^2+^ content. Moreover, ZIGIR exhibits no cytotoxicity and does not affect cell proliferation or cell function (insulin secretion) *in vitro* (12). To evaluate the potential of ZIGIR as an islet cell label *in vivo*, we delivered ZIGIR to a rat through the tail vein injection. We sacrificed the animal 30 min later and isolated the islets after a limited collagenase digestion of the pancreas. We randomly picked a piece of digested pancreatic tissue that contained both endocrine and exocrine cells. Confocal imaging of the tissue revealed exclusive ZIGIR labeling of the islet, and null fluorescence signal in the surrounding acinar tissue (**Figure 7A**). This result demonstrated the exquisite labeling specificity of ZIGIR towards pancreatic endocrine cells *in vivo*, likely attributing to the intrinsic property of high zinc level in the granular compartments of islet cells, most notably beta cells. We also compared ZIGIR labeling of isolated islets *in vitro vs*. islet labeling *in vivo* through systemic delivery. Compared to the *in vivo* ZIGIR labeling, where ZIGIR signal was seen throughout the entire islet (**Figures 7A, B**), ZIGIR labeling in the isolated islets was restricted to the outer cell layers (**Figure 7C**). The difference likely reflects the trapping of ZIGIR *in vitro* by the superficial cell layers which limit the diffusion of ZIGIR to the islet interior. In contrast, *in vivo*, ZIGIR is transported through the circulation and delivered to all islet cells through the rich vasculature within the islet. Notwithstanding the difference, for both *in vitro* and *in vivo* labeling, a subset of cells along the islet mantle exhibited lower ZIGIR signal intensity than the surrounding beta cells. These cells corresponded to mouse alpha cells and delta cells with relatively low granular Zn^2+^ content, as we previously demonstrated by the flow cytometry analysis of ZIGIR labeled mouse islet cells (12).

## Discussion

There have been growing interests in studying islet cells in their native habitat. The islet of Langerhans is a highly vascularized mini-organ amply supplied with blood circulation and oxygen (13, 14). In the pancreas, islets are encapsulated by the peri-islet capsule which separates the endocrine compartment from the exocrine cells (9, 10). In addition to sensing and responding to nutrient fluctuations in the circulation, islet cells also receive neuronal inputs that modulate their hormone release activity (15, 16). Studies on the regulated insulin secretion thus far have been largely relying on cultured beta cells or isolated islets. While these systems have yielded valuable insights into the molecular mechanisms governing stimulus-secretion coupling *in vitro*, our understanding on how islet cells behave *in vivo* and how they tune their secretion dynamics moment by moment in response to physiological cues is relatively limited. Imaging assays capable of tracking hormone release activity of islet cells in live animals would be invaluable for studying islet cell physiology, for monitoring the declining of islet cell function during diabetes progression, and for assessing the efficacy of therapeutics intended for maintaining or restoring beta cell function.

ZIMIR imaging tracks insulin/Zn^2+^ release at the cellular and subcellular resolution and has been applied to cultured cells and isolated islets (4, 17–20). To image insulin/Zn^2+^ release *in vivo*, we attempted several methods for loading ZIMIR to islet cells in the intact pancreas. The bolus dye loading is an established method for labeling neurons in the brain. In our hands, dye microinjection into the exocrine tissue was rather inefficient in labeling nearby islets, while intra-islet dye injection turned out to be challenging, likely due to the miniature size and cellular compactness of an islet. Compared to the microinjection, pancreas perfusion through the splenic artery turned out to be more effective in labeling endocrine cells with amphipathic dyes such as Cy3-C12 and ZIMIR (**Figure 3**). Among the three approaches that we have explored, the chemogenetic method of tagging the plasma membrane of islet cells with a HaloTag probe turned out to be most facile and selective, and it holds the promise for the routine application through systemic delivery in the future. After inducible expression of HaloTag protein in the transgenic mouse, we were able to achieve efficient and selective labeling of beta cells after tail vein injection of Fluo-HaloTag-2 (**Figure 5**). However, adopting this strategy to deliver ZIMIR-HaloTag to beta cells turned out to be less efficient compared with Fluo-HaloTag-2. The difference probably reflected a variation in biodistribution and pharmacokinetics (PK) of these two probes *in vivo*, such that ZIMIR-HaloTag in the circulation falls off too rapidly to react with the HaloTag protein expressed on the beta cell surface. Efforts of engineering ZIMIR derivatives to improve their PK and islet distribution are underway and, if successful, such probes are expected to boost the labeling efficiency of beta cells of the transgenic mouse.

In a pilot experiment of *in vivo* ZIMIR imaging, we observed synchronized, oscillatory insulin/Zn^2+^ releases among a cluster of mouse islet beta cells (**Figure 4**). These rhythmic oscillations exhibited a period of ~ 32 s. To our knowledge, this represents the first report of insulin release at the cellular resolution in the intact pancreas *in vivo*. Insulin oscillation has been documented in the isolated islets *in vitro*, and in the blood circulation *in vivo* (21, 22). A number of studies have shown rhythmic behavior of hormone secretion in isolated islets, with oscillating periods ranging from ~ 20 s to several minutes (21, 23, 24). Insulin oscillation is thought to be coupled with intracellular Ca^2+^ oscillation, bursting electrical activity and cellular metabolic rhythm (25). It has been proposed that oscillatory insulin release is important to normal glucose homeostasis, and disturbance of oscillations could be detrimental and play a major role in type 2 diabetes (1). The *in vivo* imaging technique that we have been developing and its future enhancements should enable investigating this important phenomenon at the cellular level and to address its regulation in live animals. The recent development of abdominal imaging window (AIW) has made it possible to track internal organs by high resolution fluorescence microscopy for a prolonged period of time (26, 27). Under favorable conditions, an animal could be imaged repeatedly up to several weeks. Even though our work described herein focuses on probe delivery as a proof of principle for the *in vivo* ZIMIR imaging, we anticipate that future probe engineering and integration with AIW would eventually lead to an imaging platform for the longitudinal monitoring of insulin release of individual islets *in vivo*. In additional to optical imaging, recent advancements in magnetic resonance imaging (MRI) of Zn^2+^ release provides yet another imaging platform for monitoring the secretory activity of cells *in vivo* (28, 29). Integration of these different imaging modalities is expected to enhance our ability to track islet beta cell function in the intact pancreas spanning a broader range of spatial and temporal scales.

Besides beta cell function, another parameter of importance to islet biology and diabetes research is beta cell mass. As we have shown here, ZIGIR represents a promising *in vivo* imaging probe of islet cell mass. ZIGIR is brightly fluorescent and can be applied at a rather low concentration to label beta cells *in vivo*. We routinely injected ZIGIR through tail vein at 10 nmol/mouse. Based on a blood volume of ~ 2 ml, this is equivalent to ~ 5 µM ZIGIR in the blood circulation immediately after injection. At this dosage, ZIGIR labeled islet cells efficiently and selectively without showing any detectable signal in the exocrine tissue (**Figure 7**). Given the bright signal we observed in the labeled islets, we speculate that even lower doses of ZIGIR can be used for the *in vivo* labeling and imaging applications. Zinc chelating agents such as dithizone (DTZ) have long been used to stain pancreatic islets (30). However, DTZ is a rather poor fluorophore for fluorescence imaging, and is limited by its cytotoxicity (31). In fact, DTZ reduces insulin secretion and causes islet cell death, and DTZ has been used as a diabetogenic agent in animal studies (32). In contrast, at micromolar concentration, ZIGIR shows no cytotoxicity, and does not affect cell proliferation or insulin secretion (12). Future molecular engineering of ZIGIR analogues emitting at longer wavelengths up to near-infrared, or incorporating radioisotopes (^18^F, ^11^C, ^125^I, etc) presents new opportunities for engineering whole body imaging platforms of interrogating islet cell physiology *in vivo*.

## Data Availability Statement

The raw data supporting the conclusions of this article will be made available by the authors, without undue reservation.

## Author Contributions

W-hL and PES designed the experiments. SC, ZH, HK, MK, ES, SX, EG, and YX conducted the experiments and analyzed the data. W-hL and SC wrote the paper. W-hL, PES, and ADS provided supervision and funding. All authors contributed to the article and approved the submitted version.

## Funding

This work was supported by grant awards from JDRF (1-SRA-2018-675-S-B to W-hL) and NIH (R01-GM132610 to W-hL, R01-DK55758 and R01-DK099110 to PES, and R01-DK095416 to ADS).

## Conflict of Interest

W-hL and EG are co-inventors of a US patent concerning ZIGIR.

The remaining authors declare that the research was conducted in the absence of any commercial or financial relationships that could be construed as a potential conflict of interest.
